# Neurosensory Symptom Complexes after Acute Mild Traumatic Brain Injury

**DOI:** 10.1371/journal.pone.0146039

**Published:** 2016-01-04

**Authors:** Michael E. Hoffer, Mikhaylo Szczupak, Alexander Kiderman, James Crawford, Sara Murphy, Kathryn Marshall, Constanza Pelusso, Carey Balaban

**Affiliations:** 1 Department of Otolaryngology, University of Miami Miller School of Medicine, Miami, Florida, United States of America; 2 University of Miami Sports Performance and Wellness Institute, Miami, Florida, United States of America; 3 Department of Neurological Surgery, University of Miami Miller School of Medicine, Miami, Florida, United States of America; 4 Neurokinetics, Inc, Pittsburgh, Pennsylvania, United States of America; 5 Department of Otolaryngology, Madigan Army Medical Center, Tacoma, Washington, United States of America; 6 Department of Otolaryngology, University of Pittsburgh, Pittsburgh, Pennsylvania United States of America; University of Florida, UNITED STATES

## Abstract

Mild Traumatic Brain Injury (mTBI) is a prominent public health issue. To date, subjective symptom complaints primarily dictate diagnostic and treatment approaches. As such, the description and qualification of these symptoms in the mTBI patient population is of great value. This manuscript describes the symptoms of mTBI patients as compared to controls in a larger study designed to examine the use of vestibular testing to diagnose mTBI. Five symptom clusters were identified: Post-Traumatic Headache/Migraine, Nausea, Emotional/Affective, Fatigue/Malaise, and Dizziness/Mild Cognitive Impairment. Our analysis indicates that individuals with mTBI have headache, dizziness, and cognitive dysfunction far out of proportion to those without mTBI. In addition, sleep disorders and emotional issues were significantly more common amongst mTBI patients than non-injured individuals. A simple set of questions inquiring about dizziness, headache, and cognitive issues may provide diagnostic accuracy. The consideration of other symptoms may be critical for providing prognostic value and treatment for best short-term outcomes or prevention of long-term complications.

## Introduction

Mild Traumatic Brain Injury (mTBI) is an increasingly important public health issue, with an especially high prevalence in sports and military populations. Efforts are underway in many labs to study this disease from a variety of approaches. These approaches range from studying the basic pathophysiology of the disorder to improving diagnostic and treatment success. Despite this work, most mTBI is diagnosed in facilities that utilize medical history and a physical exam to make the diagnosis and determine if treatment is necessary. Since complaints/symptoms remain the dominant components of diagnostic and treatment algorithms, it is critically important to describe and qualify these complaints in this patient population. In this manuscript, we describe the symptoms of mTBI patients as compared to controls in a larger study designed to examine the use of vestibular testing to diagnose mTBI.

## Materials and Methods

This study and its written informed consent material were approved independently by the following IRB's. IRB at Naval Medical Center San Diego, IRB at Madigan Army Medical Center, IRB at the University of Miami, Miller School of Medicine. Every patient who participated in this study signed a written informed consent document in a manner specified and approved by the IRB at their site.

Individuals between the ages of 18 and 45 were recruited from the emergency rooms of one civilian and two military hospitals. Individuals were eligible for inclusion in the study if they had a diagnosis of mTBI from the emergency room. They reported to the study sites at a scheduled time within six days of injury. At the study site, individuals were assessed for mTBI and the presence of any exclusion criteria. Those who were not excluded were offered participation in the study. Control subjects were recruited from staff members at the locations where the study was being conducted. These individuals were also between the ages of 18–45 and were screened to assure that they had no active medical condition and did not have any history of significant mTBI, ear or balance disorders.

**Fig 1:**
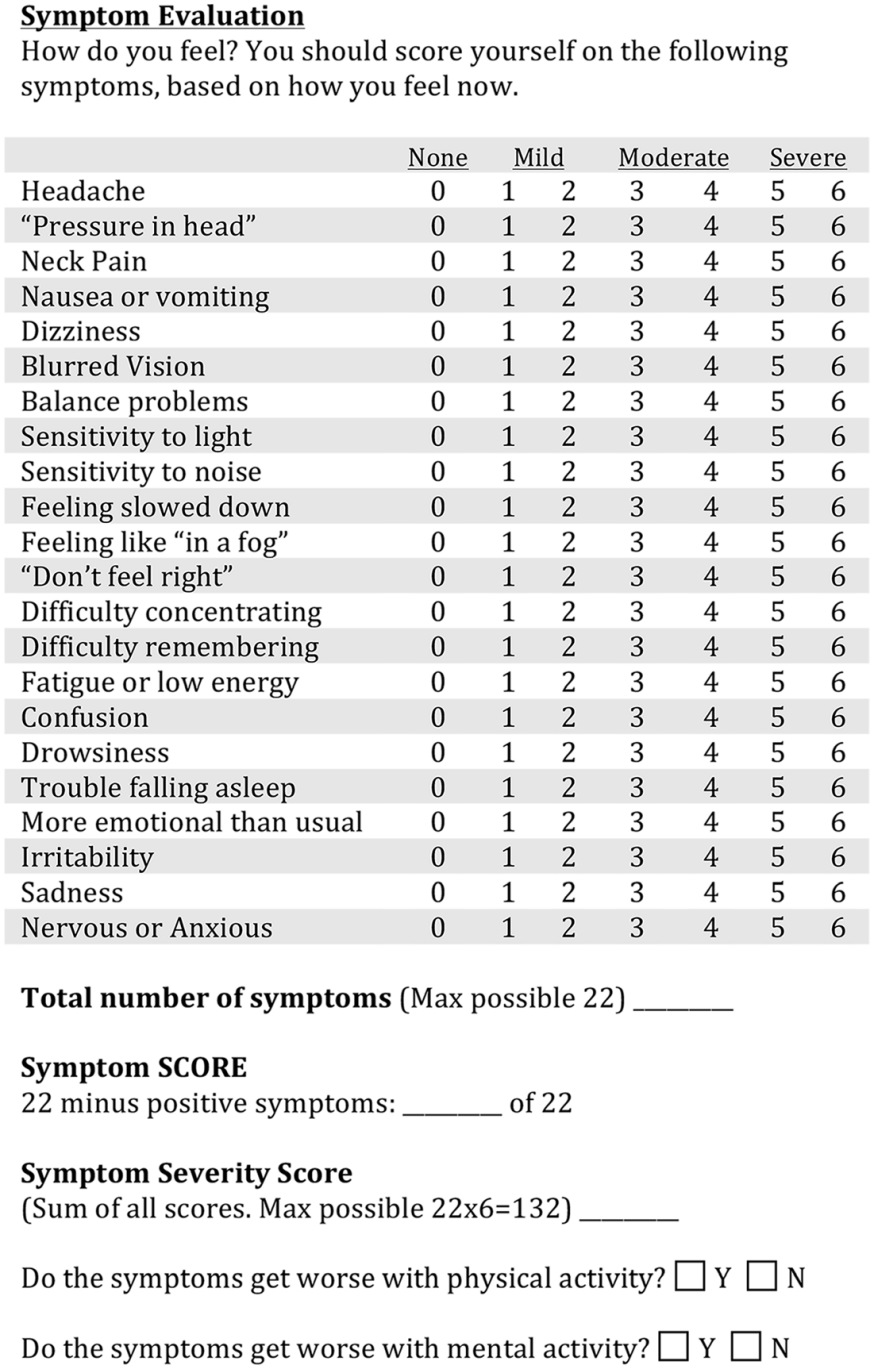
Symptom Questionnaire. Administered by an investigator in which subjects rank the symptoms on a 0–6 scale with 0 meaning “none” and 6 meaning “severe.”

**Fig 2:**
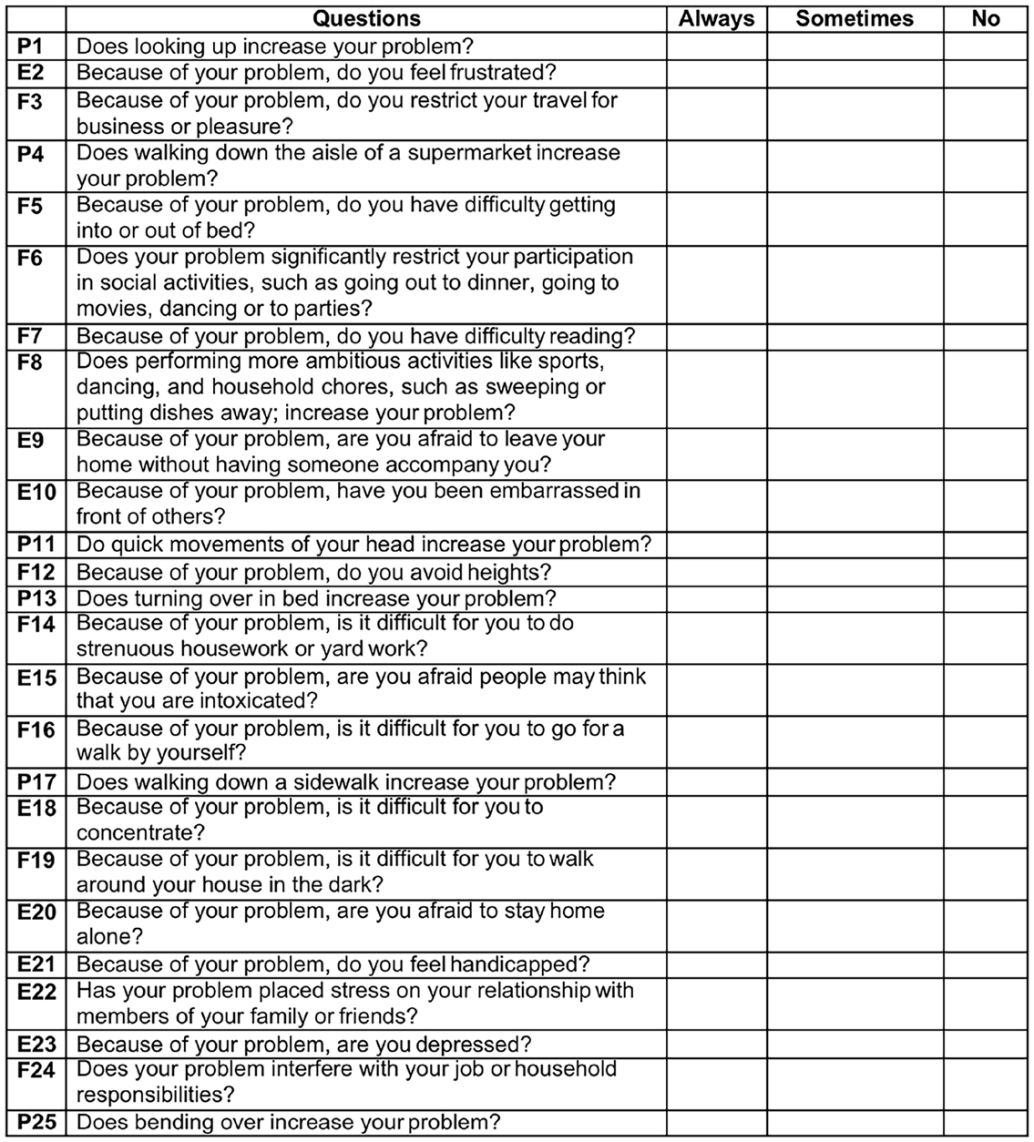
Dizziness Handicap Index (DHI). Self-administered and well normed device in which subjects ranked dizziness symptoms as always, sometimes, or never. By assigned 4 for each always, 2 for each sometimes, and 0 for each never a final ordinal score is obtained.

**Fig 3:**
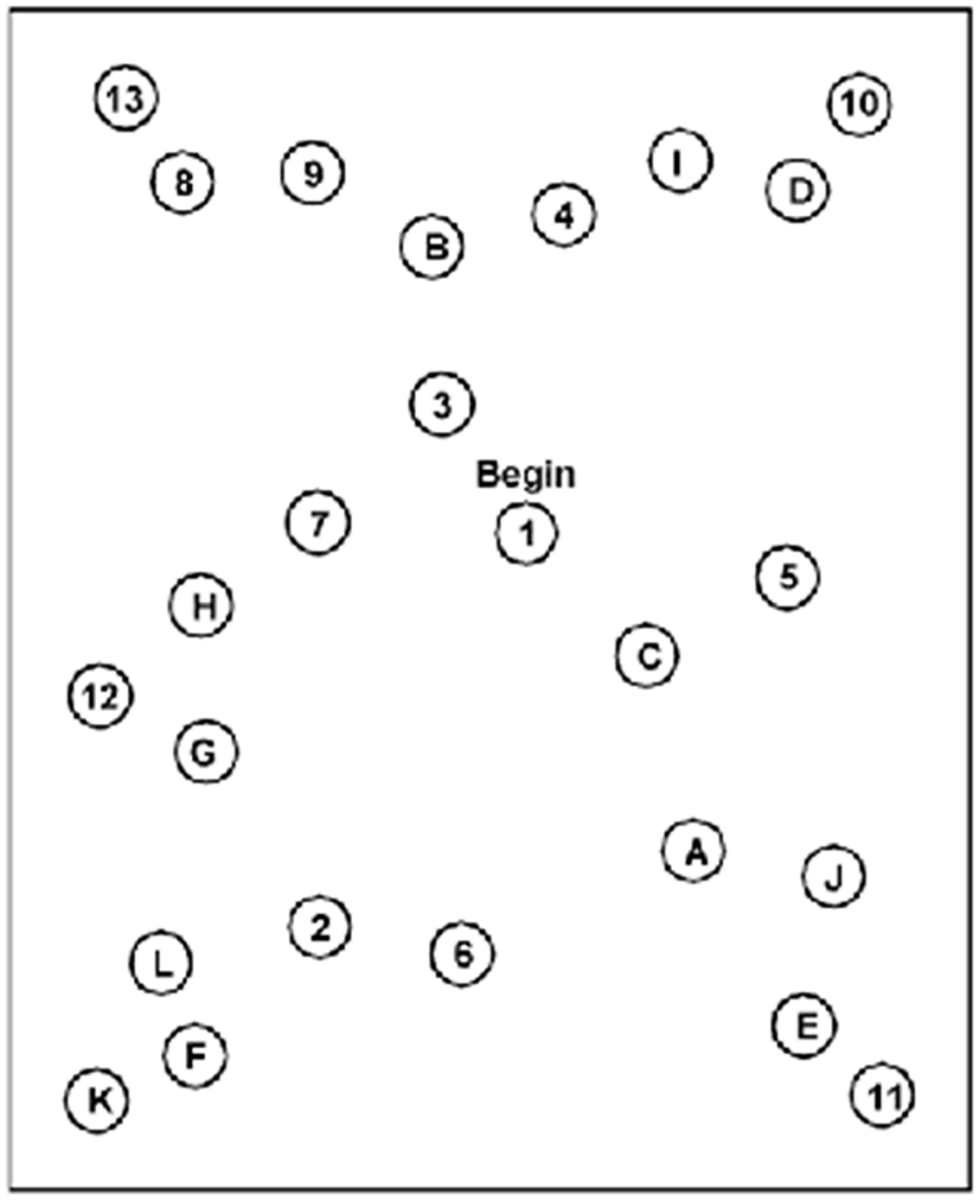
Trail Making Test B (TMTB). Self-administered pattern test going from numbers to corresponding letters in the alphabet as follows: 1→A→2→B→…→12→L→13. The pencil may not leave the paper during the test. Time to complete the tracing is recorded.

After informed consent was obtained, all participants underwent a standard assessment, which included:

1. Detailed medical history and physical
2. Investigator-administered symptom questionnaire [1]. The core of the questionnaire is a labeled magnitude estimate of the severity of 22 symptoms, on a seven-point scale from 0 (none) to 6 (severe). The adjective anchoring the scale are ‘None’ for a zero rating, ‘Mild’ for a rating of 1–2, ‘Moderate’ for a rating of 3–4, and ‘Severe’ for a rating of 5–6. Two other ‘yes-no’ questions ask if the symptoms are exacerbated by either physical or mental activity (Fig 1).
3. Dizziness Handicap Inventory (DHI) [2]. The DHI is a set of 25 questions that are answered on a three point labeled scale, ‘Always’, ‘Sometimes’ or ‘Never’ (Fig 2). Sub-scores for functional, emotional and physical issues are derived from different question subsets.
4. Trail Making Tests A and B (TMTA, TMTB). The subject is timed during performance of a paper-and-pencil test. Numbers are connected in sequence for TMTA. For TMTB, the subject connects letters and numbers in the sequence (Fig 3). Extensive subject norms have been published (e.g., Tombaugh TN (2004) Trail Making Test A and B: Normative data stratified by age and education. Archives of Clinical Neuropsychology 19: 203–214).
5. Functional Gait Assessment (FGA). Ten gait tasks are each scored on a four-point scale, ranging from three points for normal performance to zero for severe impairment. The FGA is a standard clinical test with strong cross-validation of scores by trained observers [3].
6. Rotational chair test battery for oculomotor and vestibular reflex performance. The rotational chair data are considered in detail elsewhere [4–6].

This test battery was obtained once for the controls and at three time points for the active subjects (baseline, one week post-concussion, and 14 days post-concussion).

## Results

The participants included 50 mTBI patients and 100 control subjects. The total study population was 107 (71.3%) males and 43 (28.7%) females, although the mTBI group had a higher female percentage of 42% (21/50). The mean age of the mTBI group was 26.6 years of age and the mean age of the control group was 29.4 years of age. The average time of initial presentation for those in the mTBI groups was 2.42 days (± 1.45 days SD) post-concussion.

Symptom scores for each of the 22 symptoms queried are shown in Table 1. All symptom scores showed significant group differences. It is notable that the mean ratings of symptoms for the control group were all less than 0.5, corresponding to the descriptor of ‘None’ on the questionnaire, and that the upper bounds of 95% confidence intervals do not exceed the low end of the ‘Mild’ descriptor range. The mean ratings from the mTBI group varied across the symptoms. Mean ratings in the ‘Moderate’ range (3–4) were reported for ‘Headache’, ‘Don’t feel right’ and ‘Fatigue or low energy’. Mean ratings in the ‘Mild to Moderate’ range (2–3) were reported for (in descending order) ‘Feeling slowed down’, ‘Pressure in the head’, ‘Difficulty concentrating’, ‘Drowsiness’, ‘Light sensitivity’, ‘Trouble falling asleep’, ‘Difficulty remembering’, and ‘Dizziness’. The mean ratings for the other symptoms were in the ‘Mild’ descriptor range. However, the variability in these ratings was high in the mTBI group with the 95% confidence intervals of the majority of symptoms spanning the full descriptor range from ‘None’ to ‘Severe’.

**Table 1:** Group mean and standard deviation of symptom scores.

Symptom	Control (*n* = 100)	mTBI (*n* = 50)
Headache	0.12 (0.433)	3.40 (1.678)
Pressure	0.17 (0.551)	2.56 (1.875)
Neck pain	0.15 (0.557)	1.92 (1.978)
Nausea	0.05 (0.261)	1.08 (1.368)
Dizziness	0.01 (0.100)	2.00 (1.702)
Blurred vision	0.06 (0.278)	1.32 (1.596)
Balance problems	0.05 (0.330)	1.52 (1.669)
Light sensitivity	0.11 (0.469)	2.22 (1.941)
Noise sensitivity	0.02 (0.141)	1.78 (1.753)
Feeling slowed down	0.15 (0.479)	2.68 (2.065)
Don’t feel right	0.07 (0.326)	3.04 (2.040)
Difficulty concentrating	0.07 (0.355)	2.54 (2.032)
Difficulty remembering	0.12 (0.433)	2.02 (2.075)
Fatigue or low energy	0.46 (0.858)	3.00 (1.979)
Confusion	0 (0)	1.36 (1.711)
Drowsiness	0.28 (0.726)	2.46 (2.043)
Trouble falling asleep	0.42 (1.007)	2.04 (2.267)
More emotional than usual	0.08 (0.367)	1.20 (1.641)
Irritability	0.24 (0.622)	1.82 (1.945)
Sadness	0.05 (0.261)	1.10 (1.632)
Nervous or anxious	0.18 (0.609)	1.48 (1.752)
Feeling like “in a fog”	0.03 (0.223)	1.96 (1.895)

Principal component analysis (varimax rotation with Kaiser normalization) of the data from all subjects showed that the 22 symptom scores formed five statistically uncorrelated factors (or symptom clusters) as can be seen in Table 2. A Post-Traumatic Headache/Migraine component reflects high loadings for subjective headache, a sense of “pressure in the head,” fatigue, sensitivity to sound, sensitivity to light, feeling slowed down, and ‘don’t feel right. A Dizziness/Mild Cognitive Impairment cluster (or component) reflects high contributions from subjective dizziness, blurred vision, balance problems, difficulty concentrating, difficulty remembering, and confusion. An Emotional Lability cluster dimension is anchored by large contributions from self-reported trouble falling asleep, emotional labiality, irritability, sadness and nervousness or anxiousness. A Cervicogenic Issues (Foggy-Neck Pain) component reflects high loadings for feeling ‘in a fog’, and neck pain. Nausea was anchored by that symptom alone. The Dizziness/Mild Cognitive Impairment cluster score in the mTBI group showed a significant positive correlation with the total DHI score (r = 0.65, p<0.001), as well as the functional (r = 0.69, p<0.001), physical (r = 0.48, p<0.05) and emotional (r = 0.59, p<0.001) domain scores. The other cluster scores were uncorrelated with the DHI. Neither the TMTA, the TMTB nor the FGA scores were correlated significantly with any symptom cluster score.

**Table 2:** Principal component loading of symptom scores.

	Post-traumatic Headache/Migraine	Dizziness with Mild Cognitive Impairment	Emotional Lability	Fogginess and Neck Pain	Nausea
Sensitivity to light	0.7949				
Headache	0.7323				
Sensitivity to noise	0.6691				
“Don’t feel right”	0.6293				
Pressure in head	0.6261				
Feeling slowed down	0.5804				
Drowsiness	0.5783				
Fatigue or low energy	0.5641				
Blurred vision		0.7836			
Confusion		0.7565			
Difficulty remembering		0.6564			
Balance problems		0.6033			
Difficulty concentrating		0.5820			
Dizziness		0.5773			
More emotional than usual			0.7925		
Irritability			0.7628		
Trouble falling asleep			0.7576		
Nervous or anxious			0.6982		
Sadness			0.6763		
Neck Pain				0.7937	
Feeling like “in a fog”				0.5991	
Nausea or vomiting					0.8569

**Fig 4:**
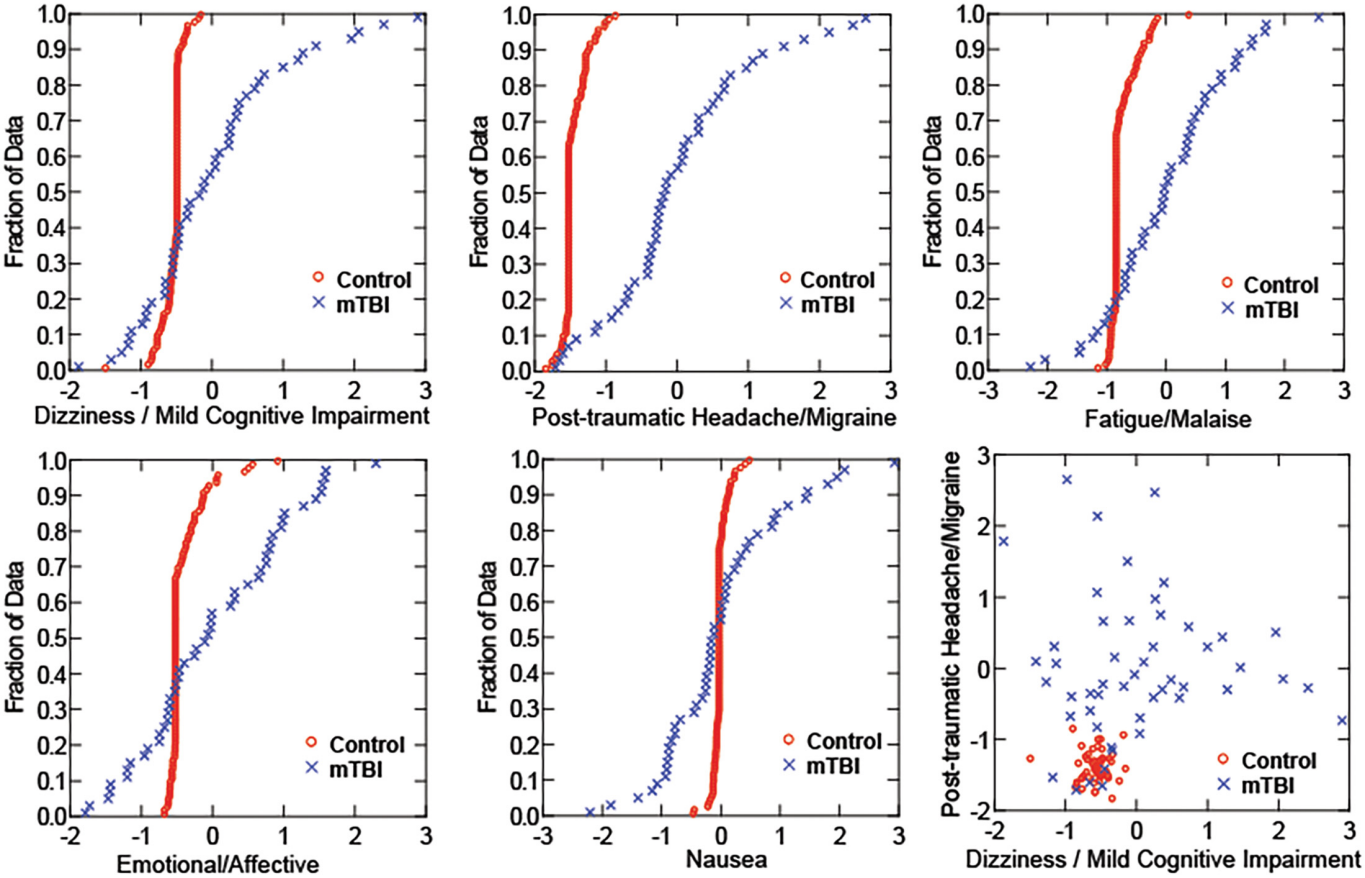
Distribution scores. Cumulative distribution scores for controls (gray) and mTBI subjects (black).

The cumulative distribution functions for the factor scores of these components (Fig 4) differ between the Control and mTBI participants, particularly for the Post-Traumatic Headache/Migraine, Dizziness/Mild Cognitive Impairment and Cervicogenic Issues components. A strongly factor positive score indicates a high ranking (moderate to severe) for the intensity of the underlying symptoms for that cluster. A negative factor score indicates that the ranking of underlying symptoms tended to be in the ‘none-to-mild’ range. It seems noteworthy that more than one-quarter of the mTBI subjects had factor scores lower than the 1% level of the Control group for Emotional Lability (13/50) and Nausea (8/50). Fewer mTBI subjects reported scores below that cutoff for Cervicogenic Issues (8/50) and Dizziness/Mild Cognitive Impairment (4/50).

**Fig 5:**
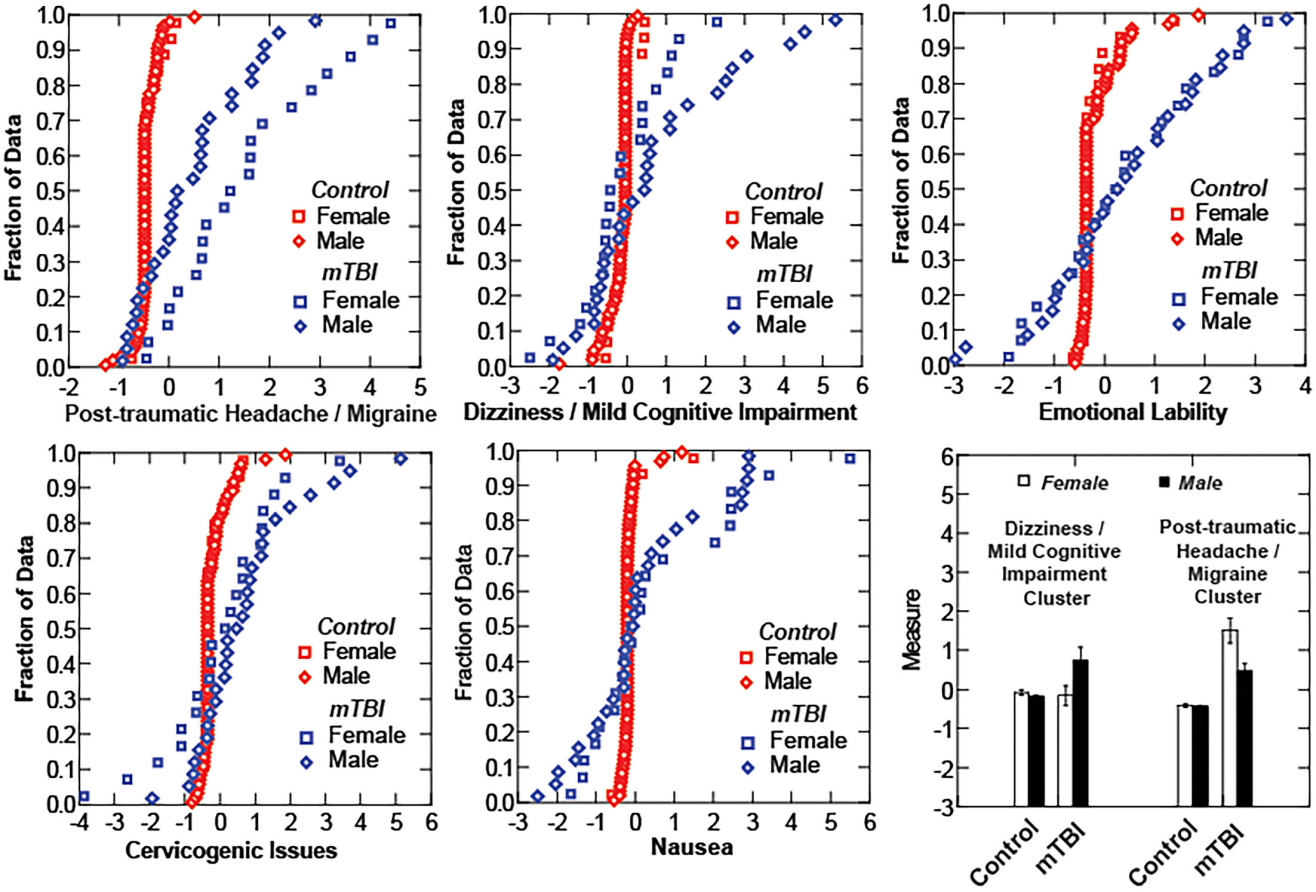
Distribution Scores. Cumulative distribution scores analyzed by sex of subject. Gray squares (female controls) and gray diamond (male controls) show on difference across symptoms clusters whereas the black squares (female mTBI) and black diamonds (male mTBI) vary across the dizziness/cognitive and headache/migraine clusters.

The relative intensity of symptom cluster factor scores showed differences between female and male subjects with mTBI (Fig 5). The cumulative distribution functions of female and male control subjects are indistinguishable for each of the components, as are the distribution functions for male and female mTBI subjects on the Emotional/Affective, Fatigue/Malaise, and Nausea component scores. However, the female subjects with mTBI reported significantly more severe Post-Traumatic Headache / Migraine cluster symptoms than their male counterparts; both female and male mTBI groups differed significantly from controls (LSD tests, p<0.001 re: the other groups). In addition, the male subjects with mTBI reported more severe Dizziness / Mild Cognitive Impairment cluster symptoms (LSD tests, p<0.01 re: the other groups) than their female counterparts (who did not differ from the controls).

Cluster analysis (k-means) indicated that the symptom component scores define six groups of study participants; means and standard deviations of component scores for each group are listed in Table 3. A Normal group included 97 Control subjects and 15/50 of the mTBI subjects. A group showing a predominance of dizzy/mild cognitive impairment symptoms included 7/50 mTBI subjects who had relatively high scores for this component, with small magnitude scores for the other components. The Nauseated group included 7/50 mTBI subjects and 2/100 Control subjects with a high nausea component score and small magnitude scores on other components; the prevalence is significantly higher in the mTBI group (Fisher’s exact test, p<0.01). Post-Traumatic Headache/Mild Cognitive Impairment, without Emotional Lability, was the defining symptom components in a group of 7/50 of the mTBI subjects. Another 5/50 reported similar levels of Post-Traumatic Headache/Mild Cognitive Impairment, but with a larger Emotional Liability factor score. Finally, Cervicogenic Issues were dominant in 9/50 of the mTBI subjects and 1/100 of the Control subjects (prevalence differs by Fisher’s exact test, p<0.01).

**Table 3:** Symptoms Cluster Factor Dimensions.

Patient Cluster (from cluster analysis)	Dizziness/ Mild Cognitive Impairment Component	Emotional/Affective Component	Cervicogenic Component	Post-traumatic Headache/ Migraine Component	Nausea Component	Number of Control/ TBI Subjects
Normal	-0.13 +/- .35	-0.23+/- 0.40	-0.21 +/- 0.38	-0.33 +/- 0.42	-0.20 +/- 0.24	**97/15**
Dizziness dominance	3.51 +/- 1.17	0.33 +/- 2.43	0.81 +/- 0.90	0.58 +/- 0.97	-0.06 +/- 1.71	**0/7**
Nauseated	0.14 +/- 1.02	1.35 +/-1.06	0.28 +/-1.14	-0.47 +/-0.40	2.50 +/- 1.33	**2/7**
Headache/ Migraine without emotional lability	-0.60 +/-1.07	-1.26 +/- 0.46	-0.27 +/- 0.95	2.55 +/- 0.84	-1.02 +/- 1.93	**0/7**
Post-traumatic headache with emotional lability	-0.38 +/-1.20	2.60 +/-1.20	-1.68 +/- 1.51	2.30 +/- 1.85	-0.11 +/- 0.73	**0/5**
Cervicogenic Issues	-0.57 +/- 1.14	0.69 +/- 1.04	2.52 +/- 1.29	0.83 +/- 0.98	-0.4) +/- 0.71	**1/9**

Three additional symptom-type items were examined. These three measures differ from the results above (which are obtained from a structure interaction with an examiner) in that they are obtained as follows: self-administered (DHI), obtained sitting still with pencil and paper [TMTA: norms 23.7 ± 7.8 seconds (SD) and TMTB: norms 49.8 ± 12.5 seconds], and the functional gait assessment [FGA, norms: 28.9 ± 1.5 (SD)] obtained by testing performance in gait and gait-related tasks. Among the mTBI subjects, 13/50 (26%) had a z score of 2 or greater on either TMT test, which is outside a 95% confidence interval for performance. Similarly, half of the mTBI subjects had FGA performance at least two standard deviations below the mean of normative data. Finally, 27/50 of the mTBI group had DHI scores of at least 30, indicating greater than mild impairment, with another 8 subjects in the mild range (between 16 and 30). Nine of the 50 subjects with mTBI were within normal limits on all three of these symptom metrics (DHI, TMTA/TMTB and FGA), 8/50 had measures outside normal limits on all three metrics, 17/50 had two measures outside normal limits, and the remaining 16/50 had a single finding outside of normal limits. Males with TBI performed significantly better on the TMTA test than females (25.9 ± 2.0 s versus 33.4 ± 2.4 s (SE), LSD test, p<0.05) but there were no gender differences in performance on the TMTB test, the total or component DHI scores, and the FGA scores.

## Discussion

The existence of group differences in individual symptom severity between the control subjects and the subjects with mTBI is expected because the symptoms being queried are part of the diagnostic criteria often used in emergency rooms to diagnose mTBI. The inherent high variability among the mTBI subject group is also an accurate reflection of presentation in the emergency room. In this sense, our results here do not differ dramatically from previous studies using symptom reports for concussion [1, 7, 8]. However, a more detailed analysis of the data gives us the ability to gain deeper insight into mTBI-related symptoms.

Principal component analysis of the symptom questionnaires from all subjects identified five uncorrelated components that represent clusters of co-varying symptoms. The component defined by high loadings for subjective headache, a sense of “pressure in the head,” sensitivity to noise, sensitivity to light, “don’t feel right”, “feeling slowed down”, drowsiness and “fatigue or low energy” is termed a ‘Post-Traumatic Headache/Migraine’ because it mirrors the symptomatic criteria for a diagnosis of “Acute post-traumatic headache attributed to mild head injury” (IHS designation 5.1.2, ICD-10 code G44.880). The Nausea component reflects primarily the severity of nausea. The Emotional Lability component captures mild precursors of post-traumatic stress, and is defined by self-ratings for “more emotional than usual”, irritability, trouble falling asleep and “nervous or anxious”. A Cervicogenic Issues component includes two features associated with whiplash injuries, neck pain and “feeling in a fog.” An unexpected finding was a symptom cluster that shared both high contributions from subjective dizziness, blurred vision and balance problems with high contributions of three symptoms of mild cognitive impairment, difficulty concentrating, difficulty remembering, and confusion. The cognitive symptoms in this Dizziness/Mild Cognitive Impairment cluster (or component) likely reflect mild disorientation secondary to compensation for balance issues, similar to interference between vestibular-related balance control and choice reaction task performance [9, 10]. The clustering of these symptoms in the acute phase is a well-known clinical correlation and provides more evidence of a link between dizziness and secondary cognitive issues.

The prevalence of negative scores among the mTBI patients, outside the range of the control group scores, for all of the clusters except Post-traumatic Headache/Migraine is quite striking. The proportion of mTBI subjects with factor scores lower than the 1% level of the Control group exceeded 25% for Emotional Lability (13/50) and Nausea (8/50). A significant, but lower proportion of mTBI subjects had symptom-based factor scores below the 1% Control group level for Cervicogenic Issues (8/50) and Dizziness/Mild Cognitive Impairment (4/50). Further investigation is needed to explore whether these low scores are clinically significant as indicators of denial (‘reverse malingering’).

The elevated values of the Post-Traumatic Headache / Migraine symptom cluster score for female subjects with mTBI is consistent with a long literature that includes the greater reporting of headache symptoms by women and, perhaps consequently, a higher prevalence of headache in women [11, 12]. Conversely, the elevated values of the Dizziness / Mild Cognitive Impairment symptom cluster score in males with mTBI is a more novel finding and suggests either that: (1) males and females use different symptom descriptions for similar underlying conditions, or that (2) there may be different patterns of injury in males and females. These findings certainly motivate detailed studies of the relationship between symptom representations and objective findings in mTBI.

Our findings of differences between females and males after mTBI and in symptom cluster analysis are not unique. There are a number literature reports that describe differences between males and females. In particular, Lucas [13] reported a higher rate of post-traumatic headache (PTH) in females who suffered mTBI when compared to male patients. Lucas also reported an increased incidence of PTH in those with pre-morbid headaches. Since women have a higher rate of pre-morbid headaches then men, the higher rate of PTH was ascribed, at least in part, to this finding. There are some literature examining differences between males and females in these types of symptom complexes. Styrke and colleagues [14] examined long-term outcomes of mTBI and found a number of differences between female and male subjects Females seemed to be significantly more likely to have symptoms compared to males, and the most prominent symptom in females was headache whereas men more commonly reported memory issues. There was also a small difference in number of symptoms noted with women exhibiting more symptoms than men. The differences noted in mTBI can even be seen with exercise alone. In a study of 45 female and 30 male athletes, Gaetz and Iverson [15] examined symptoms after exercise where head injury did not occur. Females showed improvement in the “somatic” area (sadness, irritability, etc.) while males saw improvement in concentration issues. More strikingly was that females did report an increase in symptoms in the somatic realm (headache, tingling, and dizziness) while males saw a somatic symptom increase significantly less commonly and with a significantly smaller change in severity.

The gender differences in symptom cluster reporting are based upon self-reports from symptom scale items that were designed to detect acute concussion in college football players, a predominantly male population [16]. This post-concussion assessment tool “utilizes terminology and descriptors” used commonly by the athletes to describe their symptoms. Baseline score gender differences have been reported for individual items [17]. The DHI, on the other hand, was developed and validated with data from a predominantly female population of patients presenting at an audiology clinic [18]. It had a moderate correlation with the Dizziness/Mild Cognitive Impairment symptom cluster score, but showed no gender differences in our study population. These findings motivate a broader exploration the use of gender-appropriate descriptors for self-reported symptoms to assist in the individualized diagnosis and treatment of concussion.

Given this discussion and our analysis, one might argue that simply inquiring about headache-related and dizziness-related symptom clusters can lead to a reasonably accurate diagnosis of mTBI. In fact, there is some support for this notion. Dizziness and cognitive function, as measured by oculomotor, reaction time, and vestibular tests with infrared goggles, can provide nearly 90% specificity and sensitivity from distinguishing mTBI from controls. Adding headache increases those numbers to nearly 95% [4–6]. Measuring dizziness and cognitive issues is far more accurate than inquiring about these symptoms, but devices to perform these measurements may not be available at facilities where mTBI is diagnosed. Therefore, work is underway on two fronts as follows: (1) to characterize better ways of eliciting a history of dizziness or cognitive issues, and (2) to bring devices that can measure these symptoms to the point of injury or diagnosis.

Symptoms can provide more than simply diagnosis in mTBI. Even if a set of cardinal symptoms is established to provide diagnostic accuracy, other symptoms may be critical for providing prognostic value. Still others may be the symptoms that, if treated, yield the best short-term outcomes or prevent long-term complications. Work is underway in all of these areas in our labs, as well as those of many other investigators.

## Conclusion

Symptoms experienced after an mTBI remain one of the most important tools in the diagnosis and treatment of this disorder. From our analysis, it appears that individuals with mTBI have headache, dizziness, and cognitive dysfunction far out of proportion to those without mTBI, and sleep disorders and emotional issues that are also significantly more common than non-injured individuals. A fairly simple set of questions inquiring about dizziness, headache, and cognitive issues may provide diagnostic accuracy but it remains unclear if other symptoms are more important for prognostic information or treatment planning.
